# Supplementary material for: Childhood cancer in Sweden during the COVID-19 pandemic: Temporal patterns in incidence and survival in a nationwide register-based cohort study
Source: PLoS Med. 2026 Mar 5;23(3):e1004934. doi: 10.1371/journal.pmed.1004934 (PMC12962473; doi:10.1371/journal.pmed.1004934)
Supplement: S1 Table — (PDF) [file pmed.1004934.s001.pdf]

S1 Table. Absolute number of new cancer diagnoses per quarter among children aged 0–19 years in Sweden, 2015–2022.

Year	Quarter	All cancer types	ALL	AML	Hodgkin	Non Hodgkin	CNS tumours	Non CNS solid tumours
Average 2015–2019	1Q	102.0 (94,107)	20.2 (14,31)	5.0 (3,7)	5.2 (4,7)	4.6 (2,9)	23.4 (17,32)	40.4 (32,47)
Average 2015–2019	2Q	112.6 (106,120)	22.6 (18,28)	4.6 (3,8)	8.8 (6,13)	3.4 (2,6)	30.8 (25,34)	37.8 (32,43)
Average 2015–2019	3Q	100.4 (90,111)	17.8 (15,20)	4.4 (3,7)	7.6 (6,10)	4.0 (2,7)	28.0 (20,33)	35.6 (29,40)
Average 2015–2019	4Q	98.8 (85,121)	17.6 (11,30)	3.8 (2,5)	7.2 (4,10)	2.4 (1,5)	27.2 (18,35)	36.2 (32,40)
2020	1Q	96	14	3	9	4	28	35
2020	2Q	104	13	2	7	2	29	48
2020	3Q	112	25	3	5	1	39	37
2020	4Q	104	16	3	6	5	32	41
2021	1Q	112	26	5	9	7	21	43
2021	2Q	103	25	3	9	3	27	35
2021	3Q	97	23	3	3	2	33	29
2021	4Q	107	16	2	6	2	27	50
2022	1Q	115	14	0	9	6	35	47
2022	2Q	101	17	1	5	5	22	41
2022	3Q	114	30	5	5	5	24	41
2022	4Q	99	21	1	8	4	24	37

Interval in the average 2015–2019 year represents minimum and maximum between those years.

Abbreviations: ALL, acute lymphoblastic leukemia; AML, acute myeloid leukemia; CNS, central nervous system.
