# Supplementary material for: Childhood cancer in Sweden during the COVID-19 pandemic: Temporal patterns in incidence and survival in a nationwide register-based cohort study
Source: PLoS Med. 2026 Mar 5;23(3):e1004934. doi: 10.1371/journal.pmed.1004934 (PMC12962473; doi:10.1371/journal.pmed.1004934)
Supplement: S2 Table — (PDF) [file pmed.1004934.s002.pdf]

S2 Table. Age-standardized incidence rates (ASR) of cancer per 1,000,000 children aged 0–14 years in Sweden, 2015–2022.

	Age standardized incidence rate (95% CI)			
	**Average 2015–2019**	**2020**	**2021**	**2022**
**All cancer types**	162.4 (153.9,170.9)	152 (133.6,170.3)	166.4 (147.1,185.6)	162.6 (143.3,181.8)
ALL	41.6 (37.2,46.0)	33.3 (24.6,42.1)	48.9 (38.2,59.6)	41.6 (31.6,51.6)
AML	8.7 (6.7,10.7)	3.4 (0.6,6.2)	4.5 (1.4,7.7)	2.4 (0.0,4.7)
Hodgkin	5.9 (4.3,7.4)	2.8 (0.6,5.0)	3.9 (1.2,6.6)	5.2 (2.1,8.2)
Non-hodgkin	4.4 (3.0,5.7)	3.5 (0.9,6.1)	4.6 (1.6,7.7)	6.2 (2.7,9.8)
CNS tumours	43.9 (39.5,48.3)	47.5 (37.5,57.6)	46.1 (36.1,56.1)	46.6 (36.4,56.8)
Non-CNS solid tumours	52.2 (47.3,57.0)	57.6 (46.2,69.0)	53.5 (42.5,64.4)	54.7 (43.5,66.0)

Abbreviations: ALL, acute lymphoblastic leukemia; AML, acute myeloid leukemia; CNS, central nervous system; CI, Confidence interval.
