# Supplementary material for: Childhood cancer in Sweden during the COVID-19 pandemic: Temporal patterns in incidence and survival in a nationwide register-based cohort study
Source: PLoS Med. 2026 Mar 5;23(3):e1004934. doi: 10.1371/journal.pmed.1004934 (PMC12962473; doi:10.1371/journal.pmed.1004934)
Supplement: S3 Table — (PDF) [file pmed.1004934.s003.pdf]

S3 Table. Proportion of deaths within 6 months and 1 year after a CNS tumor diagnosis among children aged 0–19 years in Sweden, 2015–2022.

	6-month mortality		1-year mortality	
	**2015–2019**	**2020-2022**	**2015–2019**	**2020-2022**
	**Dead / total No. (%)**	**Dead / total No. (%)**	**Dead / total No. (%)**	**Dead / total No. (%)**
**CNS solid tumours**	19 / 533 (3.6%)	9 / 338 (2.7%)	34 / 533 (6.4%)	15 / 338 (4.4%)
Ependymomas and choroid plexus tumor	1 / 41 (2.4%)	0 / 13 (0.0%)	1 / 41 (2.4%)	0 / 13 (0.0%)
Astrocytomas	6 / 161 (3.7%)	3 / 119 (2.5%)	12 / 161 (7.5%)	4 / 119 (3.4%)
Intracranial and intraspinal embryonal tumors	8 / 74 (10.8%)	0 / 39 (0.0%)	11 / 74 (14.9%)	0 / 39 (0.0%)
Other gliomas	0 / 12 (0.0%)	1 / 6 (16.7%)	0 / 12 (0.0%)	1 / 6 (16.7%)
Other specified or unspecified intracranial and intraspinal neoplasms	4 / 245 (1.6%)	5 / 161 (3.1%)	10 / 245 (4.1%)	10 / 161 (6.2%)

Abbreviations: CNS, central nervous system.
