# Supplementary material for: Childhood cancer in Sweden during the COVID-19 pandemic: Temporal patterns in incidence and survival in a nationwide register-based cohort study
Source: PLoS Med. 2026 Mar 5;23(3):e1004934. doi: 10.1371/journal.pmed.1004934 (PMC12962473; doi:10.1371/journal.pmed.1004934)
Supplement: S1 Fig — (PDF) [file pmed.1004934.s004.pdf]

S1 Fig. Absolute number of new cancer diagnoses per month among children aged 0–19 years in Sweden, 2015–2022.

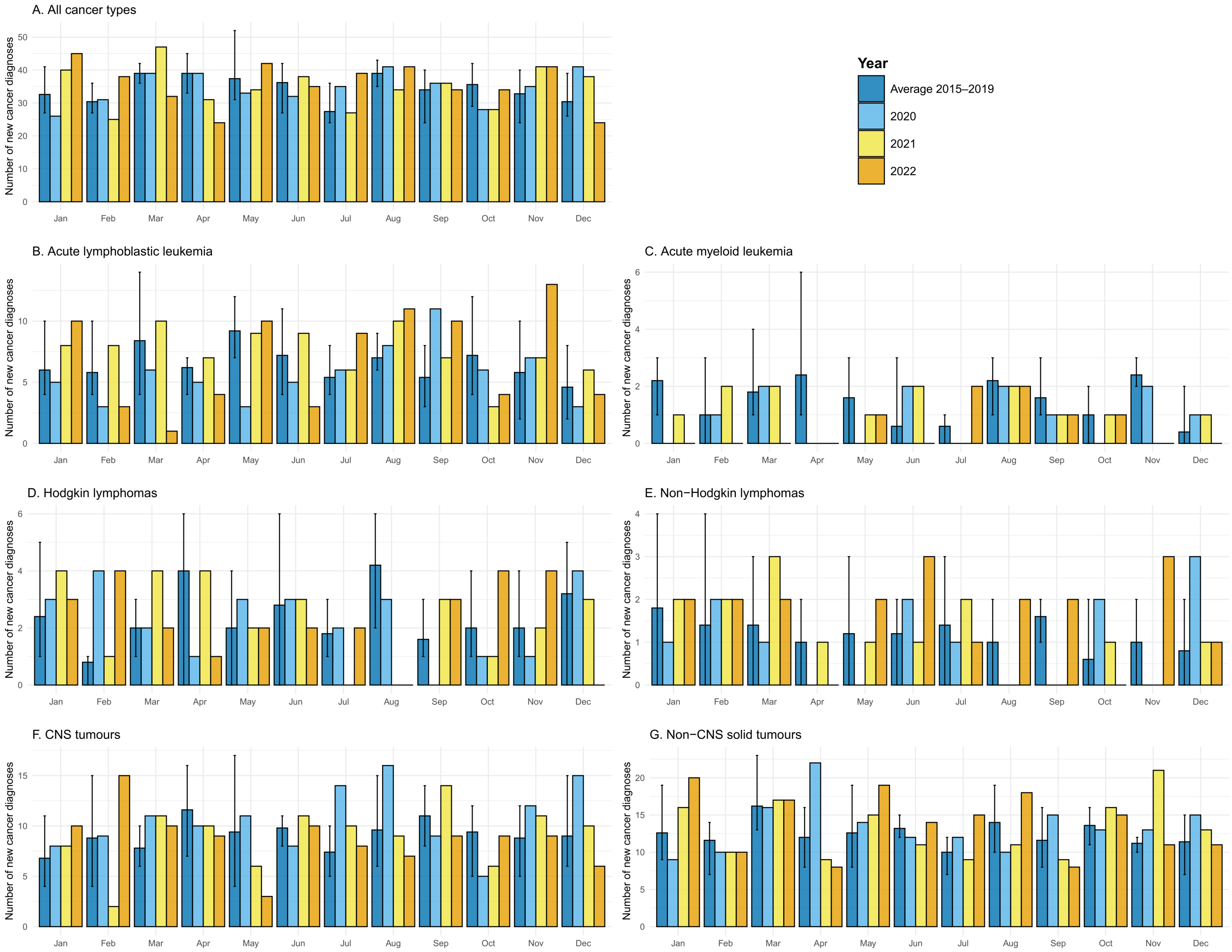

Whiskers indicate the range of values observed between 2015 and 2019.

Abbreviations: CNS, central nervous system.
