# Supplementary material for: Childhood cancer in Sweden during the COVID-19 pandemic: Temporal patterns in incidence and survival in a nationwide register-based cohort study
Source: PLoS Med. 2026 Mar 5;23(3):e1004934. doi: 10.1371/journal.pmed.1004934 (PMC12962473; doi:10.1371/journal.pmed.1004934)
Supplement: S2 Fig — (PDF) [file pmed.1004934.s005.pdf]

S2 Fig. Adjusted odds ratios (aOR) of 3-month mortality after cancer diagnosis among children aged 0–19 years in Sweden, comparing the pandemic period (2020–2022) to the pre-pandemic period (2015–2019).

Model	2015–2019<br>*Dead / total No. (%)*	2020–2022<br>*Dead / total No. (%)*	aOR ([95% CI]; p−value)
**3−month mortality**			
All cancer types	30 / 1,991 (1.5%)	17 / 1,232 (1.4%)	0.84 ([0.44,1.57]; p = 0.60)
Acute lymphoblastic leukemia	3 / 384 (0.8%)	5 / 239 (2.1%)	1.61 ([0.29,8.94]; p = 0.56)
Acute myeloid leukemia	7 / 84 (8.3%)	1 / 28 (3.6%)	0.28 ([0.01,1.88]; p = 0.27)
Hodgkin Lymphomas	0 / 144 (0.0%)	0 / 80 (0.0%)	
Non−Hodgkin Lymphomas	2 / 70 (2.9%)	1 / 46 (2.2%)	0.65 ([0.03,7.07]; p = 0.73)
CNS solid tumours	13 / 533 (2.4%)	4 / 338 (1.2%)	0.44 ([0.12,1.28]; p = 0.16)
Non−CNS solid tumours	5 / 701 (0.7%)	4 / 463 (0.9%)	1.92 ([0.42,9.86]; p = 0.40)

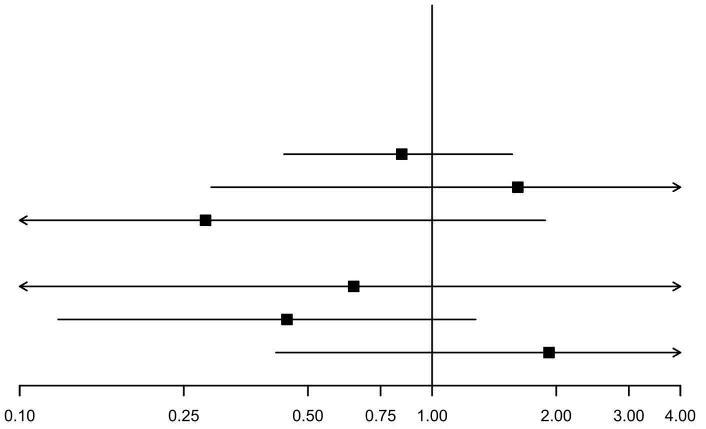

The figure is based on separate logistic regression models.

All models are adjusted by age, sex, and maternal education.

Abbreviations: CNS, central nervous system; aOR, adjusted Odds Ratio; CI, Confidence Interval; p, p−value.
