# Supplementary material for: Childhood cancer in Sweden during the COVID-19 pandemic: Temporal patterns in incidence and survival in a nationwide register-based cohort study
Source: PLoS Med. 2026 Mar 5;23(3):e1004934. doi: 10.1371/journal.pmed.1004934 (PMC12962473; doi:10.1371/journal.pmed.1004934)
Supplement: S3 Fig — (PDF) [file pmed.1004934.s006.pdf]

S3 Fig. Unadjusted odds ratios (OR) of 6-month and 1-year mortality after cancer diagnosis among children aged 0–19 years in Sweden, comparing the pandemic period (2020–2022) to the pre-pandemic period (2015–2019).

Model	2015–2019	2020–2022	OR ([95% CI]; p−value)
	Dead / total No. (%)	*Dead / total No. (%)*	
**6−month mortality**			
All cancer types	53 / 1,991 (2.7%)	28 / 1,232 (2.3%)	0.85 ([0.53,1.34]; p = 0.49)
Acute lymphoblastic leukemia	7 / 384 (1.8%)	5 / 239 (2.1%)	1.15 ([0.34,3.65]; p = 0.81)
Acute myeloid leukemia	7 / 84 (8.3%)	3 / 28 (10.7%)	1.32 ([0.27,5.15]; p = 0.70)
Hodgkin Lymphomas	0 / 144 (0.0%)	0 / 80 (0.0%)	
Non−Hodgkin Lymphomas	6 / 70 (8.6%)	2 / 46 (4.3%)	0.48 ([0.07,2.22]; p = 0.39)
CNS solid tumours	19 / 533 (3.6%)	9 / 338 (2.7%)	0.74 ([0.32,1.61]; p = 0.46)
Non−CNS solid tumours	13 / 701 (1.9%)	7 / 463 (1.5%)	0.81 ([0.30,2.00]; p = 0.66)
**1−year mortality**			
All cancer types	103 / 1,991 (5.2%)	50 / 1,232 (4.1%)	0.78 ([0.54,1.09]; p = 0.15)
Acute lymphoblastic leukemia	14 / 384 (3.6%)	5 / 239 (2.1%)	0.56 ([0.18,1.50]; p = 0.28)
Acute myeloid leukemia	11 / 84 (13.1%)	4 / 28 (14.3%)	1.11 ([0.29,3.58]; p = 0.87)
Hodgkin Lymphomas	0 / 144 (0.0%)	0 / 80 (0.0%)	
Non−Hodgkin Lymphomas	8 / 70 (11.4%)	2 / 46 (4.3%)	0.35 ([0.05,1.49]; p = 0.20)
CNS solid tumours	34 / 533 (6.4%)	15 / 338 (4.4%)	0.68 ([0.36,1.25]; p = 0.23)
Non−CNS solid tumours	32 / 701 (4.6%)	21 / 463 (4.5%)	0.99 ([0.56,1.73]; p = 0.98)

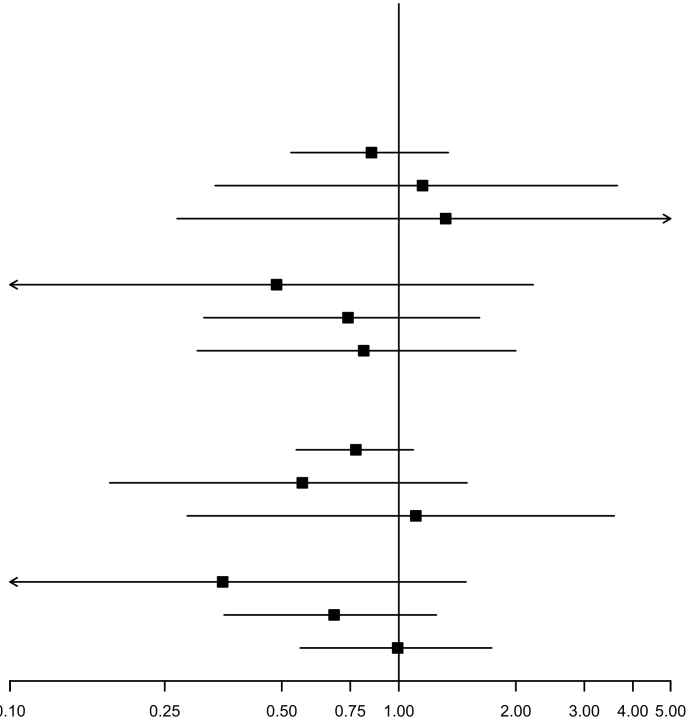

The figure is based on separate logistic regression models.

Abbreviations: CNS, central nervous system; OR, Odds Ratio; CI, Confidence Interval; p, p−value.
