# Supplementary material for: Childhood cancer in Sweden during the COVID-19 pandemic: Temporal patterns in incidence and survival in a nationwide register-based cohort study
Source: PLoS Med. 2026 Mar 5;23(3):e1004934. doi: 10.1371/journal.pmed.1004934 (PMC12962473; doi:10.1371/journal.pmed.1004934)
Supplement: S4 Fig — (PDF) [file pmed.1004934.s007.pdf]

S4 Fig. Adjusted odds ratios (aOR) of 6-month and 1-year mortality after cancer diagnosis among children aged 0–19 years in Sweden, comparing the pandemic period (2020–2022) to the pre-pandemic period (2015–2019), adjusted by paternal education.

Model	2015–2019	2020–2022	aOR ([95% CI]; p−value)
	Dead / total No. (%)	*Dead / total No. (%)*	
**6−month mortality**			
All cancer types	53 / 1,991 (2.7%)	28 / 1,232 (2.3%)	0.87 ([0.51,1.43]; p = 0.58)
Acute lymphoblastic leukemia	7 / 384 (1.8%)	5 / 239 (2.1%)	0.83 ([0.17,3.22]; p = 0.79)
Acute myeloid leukemia	7 / 84 (8.3%)	3 / 28 (10.7%)	0.71 ([0.09,3.59]; p = 0.70)
Hodgkin Lymphomas	0 / 144 (0.0%)	0 / 80 (0.0%)	
Non−Hodgkin Lymphomas	6 / 70 (8.6%)	2 / 46 (4.3%)	0.58 ([0.08,2.97]; p = 0.54)
CNS solid tumours	19 / 533 (3.6%)	9 / 338 (2.7%)	0.69 ([0.26,1.65]; p = 0.43)
Non−CNS solid tumours	13 / 701 (1.9%)	7 / 463 (1.5%)	1.20 ([0.43,3.25]; p = 0.72)
**1−year mortality**			
All cancer types	103 / 1,991 (5.2%)	50 / 1,232 (4.1%)	0.80 ([0.55,1.15]; p = 0.23)
Acute lymphoblastic leukemia	14 / 384 (3.6%)	5 / 239 (2.1%)	0.43 ([0.10,1.42]; p = 0.21)
Acute myeloid leukemia	11 / 84 (13.1%)	4 / 28 (14.3%)	0.82 ([0.16,3.16]; p = 0.78)
Hodgkin Lymphomas	0 / 144 (0.0%)	0 / 80 (0.0%)	
Non−Hodgkin Lymphomas	8 / 70 (11.4%)	2 / 46 (4.3%)	0.40 ([0.06,1.77]; p = 0.27)
CNS solid tumours	34 / 533 (6.4%)	15 / 338 (4.4%)	0.59 ([0.29,1.14]; p = 0.13)
Non−CNS solid tumours	32 / 701 (4.6%)	21 / 463 (4.5%)	1.20 ([0.66,2.15]; p = 0.54)

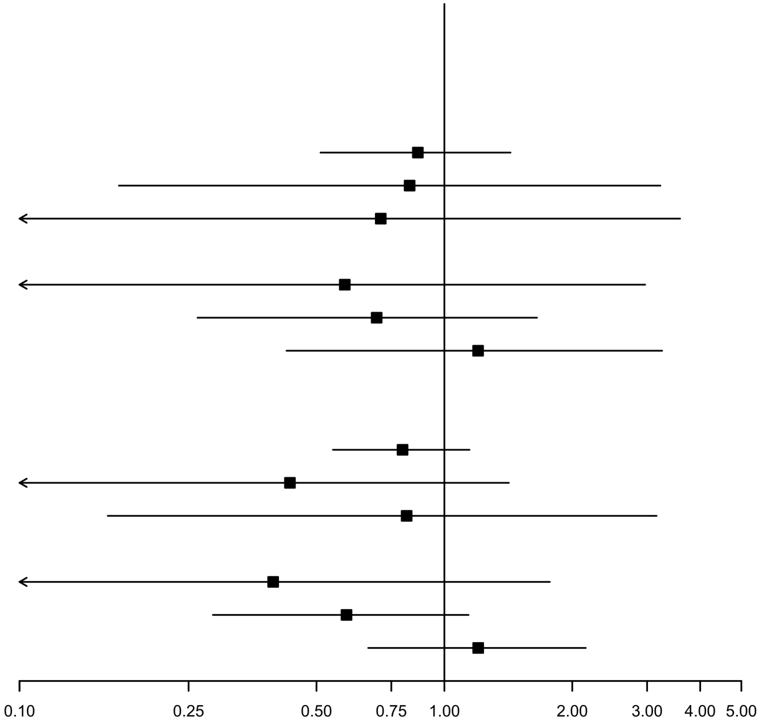

The figure is based on separate logistic regression models.

All models are adjusted by age, sex, and paternal education.

Abbreviations: CNS, central nervous system; aOR, adjusted Odds Ratio; CI, Confidence Interval; p, p−value.
