# Supplementary material for: Childhood cancer in Sweden during the COVID-19 pandemic: Temporal patterns in incidence and survival in a nationwide register-based cohort study
Source: PLoS Med. 2026 Mar 5;23(3):e1004934. doi: 10.1371/journal.pmed.1004934 (PMC12962473; doi:10.1371/journal.pmed.1004934)
Supplement: S5 Fig — (PDF) [file pmed.1004934.s008.pdf]

S5 Fig. Adjusted odds ratios (aOR) of 6-month and 1-year mortality after cancer diagnosis among children aged 0–14 years in Sweden, comparing the pandemic period (2020–2022) to the pre-pandemic period (2015–2019).

Model	2015–2019	2020–2022	aOR ([95% CI]; p−value)
	Dead / total No. (%)	*Dead / total No. (%)*	
**6−month mortality**			
All cancer types	38 / 1,370 (2.8%)	20 / 827 (2.4%)	0.76 ([0.41,1.34]; p = 0.35)
Acute lymphoblastic leukemia	6 / 348 (1.7%)	4 / 206 (1.9%)	0.60 ([0.09,2.70]; p = 0.54)
Acute myeloid leukemia	5 / 70 (7.1%)	2 / 16 (12.5%)	1.73 ([0.20,11.26]; p = 0.58)
Hodgkin Lymphomas	0 / 57 (0.0%)	0 / 24 (0.0%)	
Non−Hodgkin Lymphomas	3 / 40 (7.5%)	1 / 28 (3.6%)	0.42 ([0.02,3.87]; p = 0.49)
CNS solid tumours	16 / 378 (4.2%)	8 / 251 (3.2%)	0.64 ([0.24,1.54]; p = 0.34)
Non−CNS solid tumours	8 / 424 (1.9%)	4 / 276 (1.4%)	0.98 ([0.24,3.53]; p = 0.97)
**1−year mortality**			
All cancer types	78 / 1,370 (5.7%)	36 / 827 (4.4%)	0.74 ([0.48,1.11]; p = 0.16)
Acute lymphoblastic leukemia	12 / 348 (3.4%)	4 / 206 (1.9%)	0.36 ([0.05,1.40]; p = 0.19)
Acute myeloid leukemia	8 / 70 (11.4%)	3 / 16 (18.8%)	1.89 ([0.34,8.86]; p = 0.43)
Hodgkin Lymphomas	0 / 57 (0.0%)	0 / 24 (0.0%)	
Non−Hodgkin Lymphomas	4 / 40 (10.0%)	1 / 28 (3.6%)	0.30 ([0.01,2.43]; p = 0.32)
CNS solid tumours	31 / 378 (8.2%)	12 / 251 (4.8%)	0.52 ([0.24,1.02]; p = 0.069)
Non−CNS solid tumours	20 / 424 (4.7%)	14 / 276 (5.1%)	1.29 ([0.61,2.69]; p = 0.50)

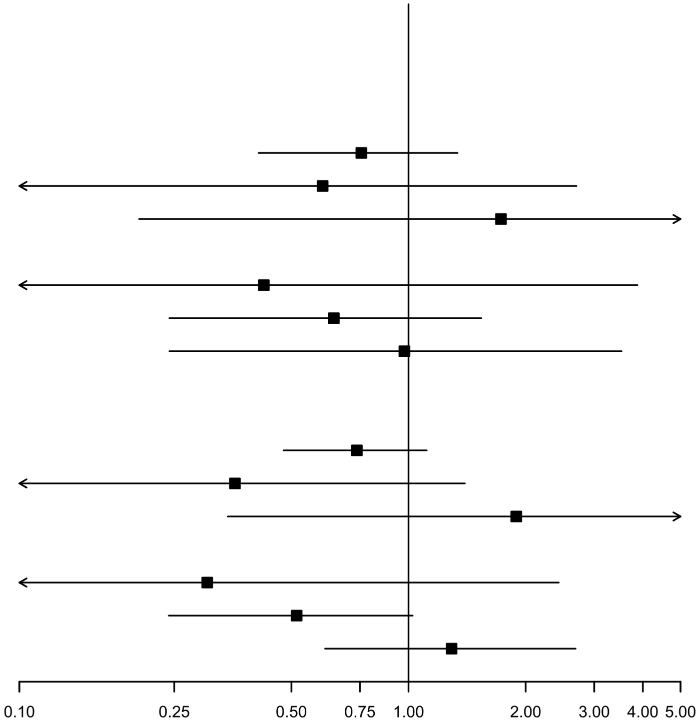

The figure is based on separate logistic regression models.

All models are adjusted by age, sex, and maternal education.

Abbreviations: CNS, central nervous system; aOR, adjusted Odds Ratio; CI, Confidence Interval; p, p−value.
