# Supplementary material for: Childhood cancer in Sweden during the COVID-19 pandemic: Temporal patterns in incidence and survival in a nationwide register-based cohort study
Source: PLoS Med. 2026 Mar 5;23(3):e1004934. doi: 10.1371/journal.pmed.1004934 (PMC12962473; doi:10.1371/journal.pmed.1004934)
Supplement: Attachment — Submitted filename: Cancer COVID IAS_response to reviewers.docx [file pmed.1004934.s012.docx]

**Authors’ note:** We sincerely thank the editorial board and the reviewers for their insightful comments and constructive suggestions, which have been invaluable in enhancing the quality of our manuscript. In response, we have carefully addressed each point raised (in blue font, below) and made the corresponding revisions. Changes are tracked in the “tracked changes” manuscript. Please note that all line numbers refer to the “tracked changes” version.

We also note that the total numbers used in the overall survival analysis have been slightly adjusted. This is because we identified that the selection of primary cancer diagnoses had initially been performed prior to applying exclusions and merging with other registers. This minor issue has now been corrected.

**COMMENTS FROM THE REVIEWERS:**

**Reviewer #1:** The authors wanted to assess the possible impact of the COVID-19 epidemic on incidence and survival of childhood cancer in Sweden. Data from pre-pandemic years 2015-2019 were compared with those from the pandemic years 2020-2022.

Although the topic is important, I am not impressed by the paper. Comments are listed below:

**Response:** We thank the reviewer for taking the time to thoroughly evaluate our manuscript. We appreciate the constructive comments and have addressed each point below, which we believe has helped strengthen the clarity and contextualization of our study.

Introduction:

Line 17, ref. 11: Data in ref. 11 are from the Oslo University Hospital. The authors should have checked NORDCAN data from all of Norway, where this tendency is not seen.

**Response:** We thank the reviewer for this insightful comment. The Norwegian study we cite indeed reflects data from Oslo University Hospital during the early months of the pandemic; we would like to note that this serves as the diagnostic center for cancer cases in the south-eastern region of Norway. We agree that it is important to clarify that some early reports, including this one, were based on local or regional rather than nationwide data—one of the knowledge gaps our study aimed to address. To this end, we have now added the following sentence to the Introduction, lines 20–21:

*“However, some of these reports included only data from one hospital or a single region rather than nationwide sources.”*

Regarding NORDCAN, we chose not to highlight these data because they are available on an annual basis at best. Since our objective was to evaluate short-term fluctuations—including potential transient drops and rebounds during specific quarters—annual incidence data do not provide the necessary temporal resolution.

Methods:

Line 72: It is right to exclude "subsequent primary neoplasms" in the survival analysis. However, the authors should have provided more absolute numbers. In Table 1, there are 1987 cancers in 2015-1019 and 1230 in 2020-2022. In Table 3, at 3-months there are 1957 cancers at risk in 2015-2019 and 1120 in 2020-2022. This means that in 2015-2019, 98% (1957/1987) of the cancers were first primary cancers, while in 2020-2022 this was 91% (1120/1230). The authors should have commented on this difference. Or maybe I misread the table, but it should be made clear what the column "at risk" means?

**Response:** We thank the reviewer for this helpful comment. Upon re-examining Table 1, we identified a typographical error. Specifically, the total numbers in the heading were incorrect. We apologize for this oversight. We have now corrected these totals: 2,069 for 2015–2019, 1,264 for 2020–2022, and 3,333 overall. The previously reported totals had been inadvertedly carried over from an earlier version of the table; we appreciate the reviewer’s careful reading.

Regarding Table 3, the numbers included in the survival analysis are 1,991 for 2015–2019 and 1,232 for 2020–2022. These correspond to 96% (1,991/2,069) and 97% (1,232/1,264) first primary cancers, respectively. The proportions are therefore similar across periods, and the small difference is compatible with random variation.

We recognize that the purpose of the “at risk” column may not have been fully clear from this Table and thank the reviewer for pointing out the need for clarification. The “at risk” column in Table 3 refers to the number of individuals who were still alive and therefore at risk of death at the specific follow-up time point (3, 6, or 12 months after diagnosis). To make this explicit, we have added the following clarification to the Results section, lines 134–137:

*“Estimates of 3-, 6-, and 12-month overall survival following childhood cancer diagnosis are presented in Table 3. The table provides cross-sectional summaries at each time point, indicating the number of individuals at risk of death at that time and the cumulative number of events that had occurred up to that time.”*

To this end, an explanatory footnote has also been added to Table 3.

In addition, to make the Table easier to read, we have added a “Start” row so that readers can clearly see the total number of individuals at risk at the beginning of follow-up. We believe that these changes have improved the readability and interpretability of the table.

Results:

Line 111: It is started correctly that … "no significant changes…". But a similar reservation is not used later.

**Response:** We thank the reviewer for this comment. In this context, the term “significant” was not intended to imply statistical significance, but rather to describe whether there were notable deviations in the observed patterns. Since we are presenting descriptive incidence data without formal statistical testing of differences between periods, we agree that this wording may be misleading.

To avoid possible confusion, we have revised the sentence to replace *“significant”* with *“substantial”*. The wording has also been checked for consistency in later sections of the manuscript.

Table 3: What does the column "at risk" refer to? At risk at what time?

**Response:** As detailed in our response to a previous comment, we have now clarified the “at risk” in the Results section and Table 3 to avoid misinterpretation.

Figure 2: Now the total number of cases is again 1987 for 2015-2019 and now 1229 for 2020-2022. So does this mean that there was no subsequent primary in 2015-2019 and one in 2020-2022?

**Response:** We thank the reviewer for this comment. As detailed in previous responses, the total number of cases is obtained by adding those “at risk” to those who died at any point during follow-up. Using this approach, the totals are 1,991 for 2015–2019 and 1,232 for 2020–2022. These totals are shown in Figure 2, which is why Table 3 did not originally include a separate “total number of cases at start” column. We recognize that this original table layout was not sufficiently clear and have now revised Table 3 to include a “total at start” column to improve clarity for the reader.

Discussion.

Lines 147-157: It should be made clear that none of these changes are statistically significant.

**Response:** We thank the reviewer for this comment. Our intention in the first paragraph of the Discussion is to provide a summary of the observed patterns in childhood cancer incidence and mortality. We did not perform formal statistical testing of differences in incidence between periods, and in the mortality analyses the confidence intervals are presented in the Results and Figures, indicating the degree of statistical uncertainty.

To ensure clarity, we have carefully reviewed the wording in the Discussion to avoid overstating the findings. In addition, as suggested in a subsequent comment, we have moved the statement regarding random variation and wide confidence intervals to the beginning of the limitations paragraph (lines 255–258) to ensure that this important limitation is clearly emphasized. We also added the following wording to that sentence (addition in bold; lines 257–258):

*“First, due to the low number of cases in some diagnostic groups, our ability to draw firm conclusions is constrained by random variation and wide confidence intervals,* ***reflecting substantial statistical uncertainty around several estimates****.”*

Lines 167-168: Well check NORDCAN. These differences may reflect changes in referral patterns rather than changes in incidence. And not statistically significant.

**Response:** We appreciate the reviewer’s point. As noted in our response to the reviewer’s earlier comment on the Introduction, the Norwegian study we cite was based on data from Oslo University Hospital, whose catchment area corresponds to the south-eastern region of Norway, during the first months of the pandemic. To clarify this, we have revised the sentence in the Discussion as follows (addition in bold; line 181):

*“Consistent with our findings, a marked decline in ALL diagnoses early in the pandemic was observed in* ***data from the south-eastern region of*** *Norway.”*

While not nationwide, such early, high-resolution reports may still be informative for identifying potential short-term diagnostic disruptions during the initial pandemic wave, especially as annual national data available through NORDCAN do not permit assessment of sub-annual fluctuations.

Regarding the reviewer’s comment on referral patterns, this might indeed be a possible explanation in the Norwegian study. However, this mechanism cannot account for our own findings, which are based on nationwide data covering all childhood cancer diagnoses in Sweden. As we discuss in the manuscript, we believe that the temporary reduction in ALL diagnoses may be more likely to reflect random variation, given the small numbers, the acute clinical presentation of ALL (which typically limits diagnostic delay), and the fact that the decline began before the pandemic had a material impact on Swedish society.

Lines 188-198: I do not follow the discussion here, as the authors started explicitly, that the decline in ALL started in February 2020, before the pandemic had any substantial impact on the Swedish society.

**Response:** We thank the reviewer for this helpful comment. We agree that the decline in ALL diagnoses beginning in February 2020 cannot plausibly be attributed to pandemic-related behavioral changes in Sweden: the first registered COVID-19 case in Sweden occurred on 31^st^ of January 2020 and the pandemic had not yet materially affected society or healthcare services at that time.

Our intention in this paragraph was not to suggest that the delayed infection hypothesis explains the pattern observed in our data, but rather to situate our findings within the broader literature on ALL etiology, which has generated considerable discussion during the pandemic. Several authors have highlighted that the COVID-19 pandemic provides a unique opportunity to examine how abrupt changes in infection exposure might influence ALL risk. In particular, Taub et al. and Borkhardt et al. described two competing theoretical expectations during the pandemic (infection-triggered increases versus trigger-removing decreases) and emphasized the importance of monitoring incidence patterns closely during this period [1,2].

In this context, we discuss the infection-related hypothesis not to attribute causality to the early 2020 ALL decline, but because the absence of such an effect in our setting is itself noteworthy. As Greaves has argued, Sweden offers a valuable natural comparison for testing infection-related hypotheses during this period, due to its lack of school closures [3], and our findings indicate that marked reductions in ALL incidence did not materialize under these conditions.

To clarify this intention, and in line with a comment from Reviewer 2, we have revised the latter part of the paragraph (lines 203–221).

Lines 199-208: repetition of Results, can be deleted.

**Response:** We thank the reviewer for this comment. We agree that the Discussion should not repeat the Results, and we have carefully reviewed this paragraph with that in mind. Our intention in this section is not to restate the findings, but to interpret the heterogeneous findings across diagnostic groups and to outline potential underlying mechanisms. To make this clearer, we have adjusted the opening sentence to explicitly link this paragraph to the preceding interpretation of the ALL findings and to signal the continuation of this discussion. The paragraph now begins (line 222):

*“For the remaining diagnostic groups, distinct trends were observed that may reflect different underlying mechanisms”*

Lines 237-239: Very important, should come first, as this is a major limitation of the study.

**Response:** We thank the reviewer for this suggestion. As noted in our response to an earlier comment, we have moved this statement to the beginning of the limitations paragraph (lines 255–258), as suggested. To further emphasize this limitation, we have also clarified the wording as follows (addition in bold; lines 257–258):

*“First, due to the low number of cases in some diagnostic groups, our ability to draw firm conclusions is constrained by random variation and wide confidence intervals****, reflecting substantial statistical uncertainty around several estimates****.”*

Line 250: The authors state the interest in assessing long-term trends in incidence and survival. So why did they end the data collection with 2022?

**Response:** We thank the reviewer for raising this important point. At the time of data extraction, the Swedish National Cancer Register contained complete and validated data only through 2022. In Sweden, there is typically a 1–2 year delay in the reporting and consolidation of data from the six regional cancer registers to the national level. As a result, when we applied for the data in 2023 and received the dataset toward the end of 2024, 2022 was the most recent year available to the National Cancer Register.

Updating this dataset would require a new application and approval process, which is time-consuming. At the same time, we believe that presenting the data currently available provides timely and valuable insights and contributes meaningfully to the ongoing discussion regarding the effects of the COVID-19 pandemic on childhood cancer incidence and survival.

Abstract.

Para starting with "overall incidence …": The data did not prove a decline. Say instead, that a tendency was seen… Same para: The last sentence can go out as these results build on so few cases that it is not worth reporting in an abstract.

**Response:** We thank the reviewer for this comment. We agree that the abstract should avoid implying a definitive decline. We have therefore revised the wording in the abstract as follows (addition in bold).

*“Overall incidence rates remained stable during the pandemic,* ***but diagnostic groups showed differing tendencies.****”*

Regarding the suggestion to remove the final sentence summarizing the mortality estimates, we respectfully believe this information is important to retain. Childhood cancer is rare, and population-based mortality estimates comparing the pandemic to the pre-pandemic period are valuable for understanding outcomes and for contributing to future evidence synthesis, including systematic reviews and meta-analyses. In addition, given the international concern about potential increases in childhood cancer mortality during the pandemic, we consider it important to report these estimates to provide a complete summary of our findings.

**Reviewer #2:** Kampitsi et al present a well-executed register-based analysis of childhood cancer incidence and short-term survival in Sweden during the COVID-19 pandemic. The authors leverage comprehensive national registry data covering 2015 to 2022 to examine whether the pandemic influenced diagnostic rates or outcomes. The study addresses an important question given inconsistent published data. Overall, the paper is of valuable, although requires some minor edits prior to publication.

**Response:** We sincerely appreciate the reviewer’s positive and encouraging assessment of our manuscript; it is gratifying to know that the reviewer found the study design and overall contribution valuable. Our point-by-point responses follow below.

*	While the study cautiously interprets observed changes in specific cancer types (e.g., the early decline and rebound in ALL), the language in parts of the Discussion may overreach. The circulation of common infections and the relationship with the development of pediatric cancers (specifically ALL) is a theory of association, without clear causal linkage.

**Response:** We thank the reviewer for this thoughtful comment. We agree that the relationship between infection patterns and childhood ALL remains a hypothesis without established causal confirmation. Our intention in the Discussion is to situate our findings within the broader scientific literature, where this hypothesis has been widely discussed, particularly in the context of the COVID-19 pandemic (1–3).

In light of this comment, and in line with a comment from Reviewer 1, we have revised the relevant paragraph to more clearly emphasize the speculative nature of these infection-related hypotheses and to acknowledge the uncertainty surrounding the underlying biological processes (lines 203–221).

*	To the prior point, although it makes sense to divide the data as pre- and during the pandemic it may oversimplify the data and negate some of the inherent natural fluctuations over time. Segregation of data from year to year in some of the presented data especially between 2015 and 2022, for example cancer disease types, would add context to some of the claims of pediatric cancer distribution changes during the pandemic.

**Response:** We thank the reviewer for this helpful suggestion. While our primary comparison focused on evaluating each pandemic year against the aggregated pre-pandemic period on a quarterly basis to capture potential shifts associated with COVID-19, we agree that visualizing the year-to-year variation within the pre-pandemic period may provide valuable additional context and help readers appreciate the natural fluctuations across years.

In response, and in line with a comment from Reviewer 3, we have added a supplementary figure (S2 Figure) showing the absolute number of new childhood cancer diagnoses per quarter for each calendar year from 2015 to 2022. This year-specific visualization complements Figure 1, where the 2015–2019 whiskers indicate the range across pre-pandemic years. We believe that including S2 Figure may help the readers to directly assess the underlying year-to-year variability that informs the aggregated pre-pandemic comparison.

*	Although there may have not been prolonged lockdown, health system deficiencies secondary to the pandemic (saturation of services, etc) are likely still are play. The largest study on the possible "indirect" impact of COVID-19 on pediatric cancer services is Graetz D, et al. Lancet Child Adolesc Health. 2021. That would be a valuable reference to cite and contextualize the data from Sweden.

**Response:** We thank the reviewer for this valuable suggestion. We agree with the reviewer’s reasoning that, even in the absence of prolonged lockdowns, indirect effects of the pandemic on childhood cancer services may still have occurred. In accordance with this comment, we have incorporated the study by Graetz et al. into the Discussion and added text to better acknowledge these potential indirect effects. Specifically, we now note that (lines 193–196):

*“Despite this lack of prolonged lockdowns, indirect health system effects of the pandemic—such as temporary strain on diagnostic and treatment services—cannot be precluded and have been reported globally in settings with both high and low COVID-19 burden (Graetz et al., 2021).”*

Furthermore, to ensure this point is appropriately reflected in the limitations, we have added the following sentence (addition in bold; line 262):

*“Therefore, the lack of increased mortality does not rule out other consequences of disrupted care (e.g., more intensive treatment, higher relapse rates****), which have been reported globally during the pandemic*** *(Graetz et al., 2021).”*

**Reviewer #3:** This manuscript presents an examination of childhood cancer rates in Sweden during the years prior to and during the COVID-19 pandemic, using registry data from national Swedish registries. My comments are focussed on the statistical analysis, which seems to be largely appropriate. Further, the conclusions drawn appear to be supported by the data. However, I do have some suggestions and comments for improvements.

**Response:** We are grateful for the reviewer’s overall positive evaluation and appreciate the acknowledgement that the analyses are appropriate and the conclusions well supported. We are thankful for the constructive statistical comments; we address each of the reviewer’s suggestions below.

1.	What dates were used to define the pandemic? It seems that years are used to define pandemic and non-pandemic time periods, but given that a pandemic was only declared in March of 2020, would it be more appropriate to consider the first quarter of 2020 as part of the non-pandemic period?

**Response:** We thank the reviewer for raising this important point. The first confirmed case of COVID-19 in Sweden occurred on 31 January 2020, and although the pandemic had not yet materially affected society or healthcare services at that time, we classified all childhood cancer diagnoses from 2020 onward as part of the pandemic period. This approach reflects that COVID-19 had already reached Sweden by early 2020 and that national public health recommendations—including guidance that could influence healthcare-seeking behavior—were introduced in March. We believe that, by reporting number of diagnoses at the quarterly level, we allow readers to identify when deviations occur, which helps mitigate the limitations of using a calendar-year boundary to define the onset of the pandemic.

Moreover, this cutoff was important for the survival and mortality analyses: children diagnosed in the first quarter of 2020 received much of their treatment during periods of 2020 that were decidedly within the pandemic, when potential disruptions to healthcare delivery could have affected outcomes; which is what we aimed to evaluate.

To make this rationale clearer in the manuscript, we have added the following sentences in the Methods (lines 70–72 and 78–79):

*“Diagnoses from January 1, 2020 onward were classified as occurring during the pandemic, as COVID-19 had already been introduced in Sweden by early 2020 and national public health recommendations were issued in March.”*

*“We assessed survival for first primary cancers diagnosed before or after January 1, 2020;* ***this definition allowed children diagnosed in early 2020 to be analyzed within the treatment context shaped by the pandemic.****”*

2.	I found Figure 1 (and Supplementary Figure 1) somewhat difficult to interpret. I think it would be easier to interpret a figure with four lines on it: one for each year 2020-2022, and one for average 2015-2019. The y-axis would be the absolute number of new cancer diagnoses; the x-axis would be 1st quarter, 2nd quarter, 3rd quarter, and 4th quarter.

**Response:** We thank the reviewer for the insightful suggestion. In response to the recommendation to use line plots, as well as the suggestion to display the pre-pandemic year separately, and in line with a suggestion from Reviewer 2, we have added a new supplementary figure (S2 Figure). This figure shows the absolute number of new childhood cancer diagnoses per quarter for each calendar year from 2015 to 2022, plotted as separate lines.

We believe this visualization allows readers to directly observe both year-to-year variability within the pre-pandemic period as well as how each pandemic year compares to that baseline. The new figure complements Figure 1, where the 2015–2019 data are aggregated and presented with whiskers representing the range across those years. Taken together, these two presentations should allow readers to evaluate both the aggregated pre-pandemic pattern and the underlying year specific fluctuations.

3.	I have two further comments on Figure 1 (which also apply to Supplementary Table 1, 2, Supplementary Figure 1). First, why is it appropriate to group together the years 2015-2019? I think it would be more appropriate to consider each of the years 2015-2019 separately, to get some idea of the changes over those years and the variability to be expected from year to year. Second, why are the years split into quarters? Why not months? Why is it necessary to split years up at all? Is there a seasonal component to cancer diagnoses that is captured through a split into quarters?

**Response:**

Regarding splitting the years: Please see our response above.

Regarding the use of quarters vs months, and the rationale for splitting years: We chose to split years into quarters to capture potential short-term or transient changes in incidence that might be obscured in annual analyses, particularly during the rapidly evolving early pandemic period, or subsequent waves. Childhood cancer is rare, and monthly resolution often results in substantial random variation; to some extent, our quarterly analyses also suffer from this, but we believe they offer a better balance between temporal resolution and statistical stability.

That being said, a monthly plot is included for completeness in the Supplement (S1 figure), but our primary analyses use quarters due to the reduced noise and clearer interpretability.

**GENERAL EDITORIAL REQUESTS:**

*	We ask every co-author listed on the manuscript to fill in a contributing author statement, making sure to declare all competing interests. If any of the co-authors have not filled in the statement, we will remind them to do so when the paper is revised. If all statements are not completed in a timely fashion this could hold up the re-review process. If new competing interests are declared later in the revision process, this may also hold up the submission. Should there be a problem getting one of your co-authors to fill in a statement we will be in contact. Please do not add or remove authors without first discussing this with the handling editor. You can see our competing interests policy here: <https://eur01.safelinks.protection.outlook.com/?url=http%3A%2F%2Fjournals.plos.org%2Fplosmedicine%2Fs%2Fcompeting-interests&data=05%7C02%7Cchristina.evmorfia.kampitsi%40ki.se%7Cb98b62bd0476438d4faa08de22151735%7Cbff7eef1cf4b4f32be3da1dda043c05d%7C0%7C0%7C638985671150319469%7CUnknown%7CTWFpbGZsb3d8eyJFbXB0eU1hcGkiOnRydWUsIlYiOiIwLjAuMDAwMCIsIlAiOiJXaW4zMiIsIkFOIjoiTWFpbCIsIldUIjoyfQ%3D%3D%7C0%7C%7C%7C&sdata=pNUH3PTJjO01C9i524I4XkSzY1ETaKR5WPRZrHrJXYQ%3D&reserved=0>.

**Response:** We thank the editors for this information. We will ensure that all co-authors complete the contributing author statements and declare any competing interests in a timely manner, as requested.

*	Please upload any figures associated with your paper as individual TIF or EPS files with 300dpi resolution at resubmission; please read our figure guidelines for more information on our requirements: <https://eur01.safelinks.protection.outlook.com/?url=http%3A%2F%2Fjournals.plos.org%2Fplosmedicine%2Fs%2Ffigures&data=05%7C02%7Cchristina.evmorfia.kampitsi%40ki.se%7Cb98b62bd0476438d4faa08de22151735%7Cbff7eef1cf4b4f32be3da1dda043c05d%7C0%7C0%7C638985671150339841%7CUnknown%7CTWFpbGZsb3d8eyJFbXB0eU1hcGkiOnRydWUsIlYiOiIwLjAuMDAwMCIsIlAiOiJXaW4zMiIsIkFOIjoiTWFpbCIsIldUIjoyfQ%3D%3D%7C0%7C%7C%7C&sdata=2IShAJIJA77hTAv5ntTcvfY7WAHhhDTDOYRyRHvLKcc%3D&reserved=0>. While revising your submission, we strongly recommend that you use PLOS's NAAS tool (<https://eur01.safelinks.protection.outlook.com/?url=https%3A%2F%2Fngplosjournals.pagemajik.ai%2Fartanalysis&data=05%7C02%7Cchristina.evmorfia.kampitsi%40ki.se%7Cb98b62bd0476438d4faa08de22151735%7Cbff7eef1cf4b4f32be3da1dda043c05d%7C0%7C0%7C638985671150361145%7CUnknown%7CTWFpbGZsb3d8eyJFbXB0eU1hcGkiOnRydWUsIlYiOiIwLjAuMDAwMCIsIlAiOiJXaW4zMiIsIkFOIjoiTWFpbCIsIldUIjoyfQ%3D%3D%7C0%7C%7C%7C&sdata=VB78eEWpcJwiHpjkAN%2FteJezK2T6kYGR4yNO0uMz%2FTM%3D&reserved=0>) to test your figure files. NAAS can convert your figure files to the TIFF file type and meet basic requirements (such as print size, resolution), or provide you with a report on issues that do not meet our requirements and that NAAS cannot fix.

After uploading your figures to PLOS's NAAS tool - <https://eur01.safelinks.protection.outlook.com/?url=https%3A%2F%2Fngplosjournals.pagemajik.ai%2Fartanalysis&data=05%7C02%7Cchristina.evmorfia.kampitsi%40ki.se%7Cb98b62bd0476438d4faa08de22151735%7Cbff7eef1cf4b4f32be3da1dda043c05d%7C0%7C0%7C638985671150379444%7CUnknown%7CTWFpbGZsb3d8eyJFbXB0eU1hcGkiOnRydWUsIlYiOiIwLjAuMDAwMCIsIlAiOiJXaW4zMiIsIkFOIjoiTWFpbCIsIldUIjoyfQ%3D%3D%7C0%7C%7C%7C&sdata=oDST7YV3n76tEWRB17PnucKbq1zegTKWGYNKtubWsjo%3D&reserved=0>, NAAS will process the files provided and display the results in the "Uploaded Files" section of the page as the processing is complete.

If the uploaded figures meet our requirements (or NAAS is able to fix the files to meet our requirements), the figure will be marked as "fixed" above. If NAAS is unable to fix the files, a red "failed" label will appear above.

When NAAS has confirmed that the figure files meet our requirements, please download the file via the download option, and include these NAAS processed figure files when submitting your revised manuscript.

**Response:** We thank the editors for this guidance. All figures have now been prepared and uploaded in TIF format, as requested.

*	Please ensure that the paper adheres to the PLOS Data Availability Policy (see <https://eur01.safelinks.protection.outlook.com/?url=http%3A%2F%2Fjournals.plos.org%2Fplosmedicine%2Fs%2Fdata-availability&data=05%7C02%7Cchristina.evmorfia.kampitsi%40ki.se%7Cb98b62bd0476438d4faa08de22151735%7Cbff7eef1cf4b4f32be3da1dda043c05d%7C0%7C0%7C638985671150395673%7CUnknown%7CTWFpbGZsb3d8eyJFbXB0eU1hcGkiOnRydWUsIlYiOiIwLjAuMDAwMCIsIlAiOiJXaW4zMiIsIkFOIjoiTWFpbCIsIldUIjoyfQ%3D%3D%7C0%7C%7C%7C&sdata=JJJspfdwUyaMHp7DkpgzcCUFMBnCIus0OZ07WLZjX0w%3D&reserved=0>), which requires that all data underlying the study's findings be provided in a repository or as Supporting Information. For data residing with a third party, authors are required to provide instructions with contact information (web or email address) for obtaining the data. Please note that a study author cannot be the contact person for the data. PLOS journals do not allow statements supported by "data not shown" or "unpublished results." For such statements, authors must provide supporting data or cite public sources that include it.

**Response:** We thank the editors for this information. The required information is already provided in our Data Availability statement, which specifies how eligible researchers can obtain the underlying data from the relevant Swedish authorities.

*	We expect all researchers with submissions to PLOS in which author-generated code underpins the findings in the manuscript to make all author-generated code available without restrictions upon publication of the work. In cases where code is central to the manuscript, we may require the code to be made available as a condition of publication. Authors are responsible for ensuring that the code is reusable and well documented. Please make any custom code available, either as part of your data deposition or as a supplementary file. Please add a sentence to your data availability statement regarding any code used in the study, e.g. "The code used in the analysis is available from Github [URL] and archived in Zenodo [DOI link]" Please review our guidelines at <https://eur01.safelinks.protection.outlook.com/?url=https%3A%2F%2Fjournals.plos.org%2Fplosmedicine%2Fs%2Fmaterials-software-and-code-sharing&data=05%7C02%7Cchristina.evmorfia.kampitsi%40ki.se%7Cb98b62bd0476438d4faa08de22151735%7Cbff7eef1cf4b4f32be3da1dda043c05d%7C0%7C0%7C638985671150412279%7CUnknown%7CTWFpbGZsb3d8eyJFbXB0eU1hcGkiOnRydWUsIlYiOiIwLjAuMDAwMCIsIlAiOiJXaW4zMiIsIkFOIjoiTWFpbCIsIldUIjoyfQ%3D%3D%7C0%7C%7C%7C&sdata=xDtN2e3D4Z03WN9lQCrOJXx51EsgFSIHlg8cfpnomVc%3D&reserved=0> and ensure that your code is shared in a way that follows best practice and facilitates reproducibility and reuse. Because Github depositions can

be readily changed or deleted, we encourage you to make a permanent DOI'd copy (e.g. in Zenodo) and provide the URL.

**Response:** We thank the editors for this information and fully support PLOS’ commitment to transparency and reproducibility. We have now made the code used in the analysis publicly available and added a corresponding sentence and link to the Data Availability statement.

**FORMATTING - GENERAL**

*	Abstract: Please structure your abstract using the PLOS Medicine headings (Background, Methods and Findings, Conclusions). Please combine the Methods and Findings sections into one section.

**Response:** The abstract is formatted as instructed.

*	At this stage, we ask that you include a short, non-technical Author Summary of your research to make findings accessible to a wide audience that includes both scientists and non-scientists. The Author Summary should immediately follow the Abstract in your revised manuscript. This text is subject to editorial change and should be distinct from the scientific abstract. Ideally each sub-heading should contain 2-3 single sentence, concise bullet points containing the most salient points from your study. In the final bullet point of 'What Do These Findings Mean?', please include the main limitations of the study in non-technical language. Please see our author guidelines for more information: <https://eur01.safelinks.protection.outlook.com/?url=https%3A%2F%2Fjournals.plos.org%2Fplosmedicine%2Fs%2Frevising-your-manuscript%23loc-author-summary&data=05%7C02%7Cchristina.evmorfia.kampitsi%40ki.se%7Cb98b62bd0476438d4faa08de22151735%7Cbff7eef1cf4b4f32be3da1dda043c05d%7C0%7C0%7C638985671150428005%7CUnknown%7CTWFpbGZsb3d8eyJFbXB0eU1hcGkiOnRydWUsIlYiOiIwLjAuMDAwMCIsIlAiOiJXaW4zMiIsIkFOIjoiTWFpbCIsIldUIjoyfQ%3D%3D%7C0%7C%7C%7C&sdata=sfz00%2BHh6FVw%2BlA%2F07D%2F0S0s5Ktv9KHUTvj81Ns6a4c%3D&reserved=0>.

**Response:** An Author Summary is now included in the manuscript:

***Why Was This Study Done?***

- *Childhood cancer requires timely diagnosis and uninterrupted treatment, and there have been concerns that the COVID-19 pandemic might have disrupted this care.*
- *Reports from several countries have reported heterogeneous findings about whether fewer children were diagnosed with cancer during the pandemic; research about survival during this period is particularly scarce.*
- *Sweden kept schools and most of society open, offering a unique setting to understand how the pandemic may have affected incidence and survival of childhood cancer.*

***What Did the Researchers Do and Find?***

- *We examined all cancers diagnosed in children and adolescents in Sweden from 2015 to 2022, using nationwide health registers (3,333 children and adolescents).*
- *We compared the number of new diagnoses and survival up to one year after diagnosis during the pandemic years (2020–2022) with the pre-pandemic years (2015–2019).*
- *Overall, childhood cancer diagnoses did not decrease during the pandemic.*
- *Some cancers showed transient increases or decreases, but these changes did not translate into worse survival outcomes.*

***What Do These Findings Mean?***

- *Childhood cancer care in Sweden remained resilient during the pandemic, with no evidence that delays or disruptions led to poorer short-term survival.*
- *Temporary changes in diagnoses of specific cancer types likely reflect normal fluctuations rather than lasting effects of the pandemic.*
- *The findings highlight the value of strong, adaptable healthcare systems in protecting essential services during global crises.*
- *Longer-term monitoring is needed to understand whether incidence patterns or outcomes change in the post-pandemic era.*

*	Please express the main results with 95% CIs as well as p values. When reporting p values please report as p<0.001 and where higher as the exact p value p=0.002, for example. Throughout, suggest reporting statistical information as follows to improve clarity for the reader "22% (95% CI [13%,28%]; p</=)". Please be sure to define all numerical values at first use.

**Response:** Results have been expressed as requested.

*	Please include page numbers and line numbers in the manuscript file. Use continuous line numbers (do not restart the numbering on each page).

**Response:** Both page numbers and line numbers are now included in the manuscript file.

*	Please cite the reference numbers in square brackets. Citations should precede punctuation.

**Response:** Citations have been updated as requested.

FIGURES AND TABLES

*	Please provide titles and legends for all figures and tables (including those in Supporting Information files).

**Response:** Titles and legends are provided in the manuscript for all Figures and Tables, including Supplemental Information.

*	Please define all abbreviations used in each figure/table (including those in Supporting Information files).

**Response:** Abbreviations used in each figure/table have been defined as requested.

*	Please consider avoiding the use of red and green in order to make your figure more accessible to those with color blindness.

**Response:** We confirm that the figures already avoid red-green contrasts and meet accessibility recommendations.

**SUPPLEMENTARY MATERIAL**

*	Please note that supplementary material will be posted as supplied by the authors. Therefore, please amend it according to the relevant comments outlined here.

**Response:** We thank the editorial team for this information and confirm we have amended the supplement according to the comments below.

*	Please cite your Supporting Information as outlined here: <https://eur01.safelinks.protection.outlook.com/?url=https%3A%2F%2Fjournals.plos.org%2Fplosmedicine%2Fs%2Fsupporting-information&data=05%7C02%7Cchristina.evmorfia.kampitsi%40ki.se%7Cb98b62bd0476438d4faa08de22151735%7Cbff7eef1cf4b4f32be3da1dda043c05d%7C0%7C0%7C638985671150447679%7CUnknown%7CTWFpbGZsb3d8eyJFbXB0eU1hcGkiOnRydWUsIlYiOiIwLjAuMDAwMCIsIlAiOiJXaW4zMiIsIkFOIjoiTWFpbCIsIldUIjoyfQ%3D%3D%7C0%7C%7C%7C&sdata=4pH36080QuaScZCbXYwjcHPO7MdW975P64RgRbNVtew%3D&reserved=0>

**Response:** Supporting Information is now cited according to the recommendations.

**REFERENCES**

*	PLOS uses the numbered citation (citation-sequence) method and first six authors, et al.

**Response:** We confirm our manuscript adheres to PLOS referencing guidelines.

*	Please ensure that journal name abbreviations match those found in the National Center for Biotechnology Information (NCBI) databases (<https://eur01.safelinks.protection.outlook.com/?url=http%3A%2F%2Fwww.ncbi.nlm.nih.gov%2Fnlmcatalog%2Fjournals&data=05%7C02%7Cchristina.evmorfia.kampitsi%40ki.se%7Cb98b62bd0476438d4faa08de22151735%7Cbff7eef1cf4b4f32be3da1dda043c05d%7C0%7C0%7C638985671150469217%7CUnknown%7CTWFpbGZsb3d8eyJFbXB0eU1hcGkiOnRydWUsIlYiOiIwLjAuMDAwMCIsIlAiOiJXaW4zMiIsIkFOIjoiTWFpbCIsIldUIjoyfQ%3D%3D%7C0%7C%7C%7C&sdata=r3j7la3fhoCNvMgfmd0FaOub%2BgWfzNo%2FkX4p4Ju88Oo%3D&reserved=0>), and are appropriately formatted and capitalised.

**Response:** We confirm journal name abbreviations are handled as advised.

*	Where website addresses are cited, please include the complete URL and specify the date of access (e.g. [accessed: 12/06/2023]).

**Response:** We confirm website addresses are cited as requested.

*	Please also see <https://eur01.safelinks.protection.outlook.com/?url=https%3A%2F%2Fjournals.plos.org%2Fplosmedicine%2Fs%2Fsubmission-guidelines%23loc-references&data=05%7C02%7Cchristina.evmorfia.kampitsi%40ki.se%7Cb98b62bd0476438d4faa08de22151735%7Cbff7eef1cf4b4f32be3da1dda043c05d%7C0%7C0%7C638985671150489602%7CUnknown%7CTWFpbGZsb3d8eyJFbXB0eU1hcGkiOnRydWUsIlYiOiIwLjAuMDAwMCIsIlAiOiJXaW4zMiIsIkFOIjoiTWFpbCIsIldUIjoyfQ%3D%3D%7C0%7C%7C%7C&sdata=LFv9poZQgq8KYwxlf0guk8ldIcT1R6x%2Fv5k7vCmzOvA%3D&reserved=0> for further details on reference formatting.

**Response:** We thank the editorial team for this information. We confirm our manuscript adheres to PLOS reference formatting guidelines.

**STUDY TYPE-SPECIFIC REQUESTS**

*	Abstract: Please include the study design, population and setting, number of participants, years during which the study took place (enrollment and follow up), length of follow up, and main outcome measures.

**Response:** We confirm that the abstract includes the requested information.

*	Please ensure that the study is reported according to the RECORD guideline (available from <https://eur01.safelinks.protection.outlook.com/?url=https%3A%2F%2Fwww.record-statement.org%2F&data=05%7C02%7Cchristina.evmorfia.kampitsi%40ki.se%7Cb98b62bd0476438d4faa08de22151735%7Cbff7eef1cf4b4f32be3da1dda043c05d%7C0%7C0%7C638985671150506248%7CUnknown%7CTWFpbGZsb3d8eyJFbXB0eU1hcGkiOnRydWUsIlYiOiIwLjAuMDAwMCIsIlAiOiJXaW4zMiIsIkFOIjoiTWFpbCIsIldUIjoyfQ%3D%3D%7C0%7C%7C%7C&sdata=x%2FVlscryyydwhAUyDEd8XyC6GR64uUKokU9CfgvWnLA%3D&reserved=0>) and include the completed checklist as Supporting Information. Please add the following statement, or similar, to the Methods: "This study is reported as per the Reporting of Studies Conducted using Observational Routinely-Collected Data (RECORD) guideline (S1 Checklist)." When completing the checklist, please use section and paragraph numbers, rather than page numbers.

**Response:** We confirm that the study is reported according to the RECORD guideline and the completed checklist has been included as Supporting Information (S1 Checklist). The requested statement has been added to the Methods.

*	For all observational studies, in the manuscript text, please indicate: (1) the specific hypotheses you intended to test, (2) the analytical methods by which you planned to test them, (3) the analyses you actually performed, and (4) when reported analyses differ from those that were planned, transparent explanations for differences that affect the reliability of the study's results. If a reported analysis was performed based on an interesting but unanticipated pattern in the data, please be clear that the analysis was data driven.

**Response:** We confirm that the study is reported as instructed.

*	Please state in the Methods section whether the study had a prospective protocol or analysis plan. If a prospective analysis plan (from your funding proposal, IRB or other ethics committee submission, study protocol, or other planning document written before analyzing the data) was used in designing the study, please include the relevant document(s) with your revised manuscript as a Supporting Information file to be published alongside your study and cite it in the Methods section. A legend for this file should be included at the end of your manuscript. If no such document exists, please make sure that the Methods section transparently describes when analyses were planned, and when/why any data-driven changes to analyses took place. Changes in the analysis, including those made in response to peer review comments, should be identified as such in the Methods section of the paper, with rationale.

**Response:** We confirm that the Methods section already includes a reference to the prospective study protocol, which was registered on clinicaltrials.gov prior to data analysis (NCT06482281). Deviations from this protocol have been noted in the Methods, lines 93–95:

*“Two deviations from the registered protocol occurred: the 6-month survival analysis was added post hoc, and the originally planned inclusion of Danish data was not implemented due to data access constraints.”*

**REFERENCES**

1.	Taub JW, Ge Y, Xavier AC. COVID-19 and childhood acute lymphoblastic leukemia. Pediatr Blood Cancer. Jul 2020;67(7):e28400. doi:10.1002/pbc.28400

2.	Borkhardt A, Schuz J, Trubenbach C, Wellbrock M, Spix C, Erdmann F. Temporal changes of the incidence of childhood B-cell precursor acute lymphoblastic leukaemia in Germany during the COVID-19 pandemic. Leukemia. Dec 2022;36(12):2908-2911. doi:10.1038/s41375-022-01730-x

3.	Greaves M. COVID-19 and childhood acute lymphoblastic leukemia. Pediatr Blood Cancer. Dec 2020;67(12):e28481. doi:10.1002/pbc.28481
