# Supplementary material for: Childhood cancer in Sweden during the COVID-19 pandemic: Temporal patterns in incidence and survival in a nationwide register-based cohort study
Source: PLoS Med. 2026 Mar 5;23(3):e1004934. doi: 10.1371/journal.pmed.1004934 (PMC12962473; doi:10.1371/journal.pmed.1004934)
Supplement: Attachment — Submitted filename: Cancer COVID IAS_R2_authors response.docx [file pmed.1004934.s013.docx]

**Authors’ note:** We are grateful to the editors and reviewers for their careful evaluation of our revised manuscript and for their constructive and insightful comments. We are pleased that the paper is considered suitable for publication pending these final revisions. We have addressed all remaining points raised (in blue font, below) and made the corresponding revisions to the manuscript. All changes are tracked in the “tracked changes” version.

**COMMENTS FROM REVIEWERS:**

**Reviewer #1:** none

**Response:** We thank again the reviewer for their time and consideration of our manuscript.

**Reviewer #2:** The authors have comprehensively addressed the comments from the first submission.

**Response:** We thank the reviewer for their positive assessment of our revisions and for their constructive input during the review process, which helped strengthen the manuscript.

**Reviewer #3:** I thank the authors for their responses to my comments.

My view is that Figure 1 in the paper should be replaced by a variant of Figure S2, which I believe displays the salient information about variability between years more clearly than Figure 1. Suggestions for further improvements to this figure are to use separate plotting symbols for each year. It may also be helpful to colour the 2015-2019 years in various shades of grey and overlay the average 2015-2019 counts using a thicker black line.

**Response:** We thank the reviewer for their careful re-evaluation of the manuscript and for the helpful final suggestion. In line with this comment and the editors’ recommendation, we have replaced Figure 1 with a revised variant of Figure S2 that more clearly displays year-to-year variability. The 2015-2019 years are plotted with different symbols and in different shades of grey, and a separate line for the average 2015-2019 counts has also been plotted. As a result, Figure S2 has been removed from the Supplement, as it is now redundant.

**REQUESTS FROM EDITORS:**

GENERAL

* Please confirm that your title complies with to PLOS Medicine's style. Your title must be nondeclarative and not a question. It should begin with main concept if possible. "Effect of" (or “Impact of”) should be used only if causality can be inferred, i.e., for an RCT. Please place the study design ("A randomized controlled trial," "A retrospective study," "A modelling study," etc.) in the subtitle (ie, after a colon).

**Response:** We thank the editors for this guidance. We have revised the title to comply with PLOS Medicine style by removing causal language, beginning with the main concept, and including the study design in the subtitle. The revised title now reads:
*“Childhood cancer in Sweden during the COVID-19 pandemic: Temporal patterns in incidence and survival in a nationwide register-based cohort study”*

* Statistical reporting: Please revise throughout the manuscript, including tables and figures.

- Please report statistical information as follows to improve clarity for the reader, ""XX% (95% CI [XX,YY]; p</=)"".

- Please separate upper and lower bounds with commas instead of hyphens as the latter can be confused with reporting of negative values.

- Please repeat statistical definitions (HR, CI etc.) for each set of parentheses.

**Response:** We have revised the statistical reporting throughout the abstract, manuscript, tables, and figures to comply with PLOS Medicine style as requested.

* Please ensure that all abbreviations are defined at first use throughout the text (including statistical abbreviations).

**Response:** We have reviewed the manuscript and confirm that all abbreviations are defined at first use throughout the text. Abbreviations are also defined in all figure legends and table footnotes.

* Please ensure that tables and figures, including those in supplementary files, are appropriately referenced in the main text. We noted that, starting with Figure S2/Figure S3, the referencing is incorrect.

**Response:** We thank the editors for pointing this out. We have ensured that all tables and figures are referenced appropriately in the main text.

* Please review your text for claims of novelty or primacy (e.g. 'for the first time' or ‘novel’) and remove this language.

**Response:** We have reviewed the text accordingly and did not identify any claims of novelty or primacy.

* Please confirm that any use of statistical terms (such as trend or significant) are supported by the data, and if not please remove them. The term trend should be used only when the test for trend has been conducted.

**Response:** We have checked this and confirm that any use of statistical terms is now always supported by the data. To this end, we have replaced the term “trend” with neutral descriptive language (“patterns”, “fluctuations”).

* Please provide titles and legends for all figures and tables (including those in Supporting Information files).

**Response:** We have ensured that all tables and figures, including supplementary files, are appropriately referenced.

* Please define all acronyms used in each figure or table in the corresponding legend.

**Response:** We have ensured that all acronyms used in figures and tables are defined in the corresponding legend.

* Please confirm that you used patient-centered language. Please note that patient-centered language is constructed with the use of post-modified nouns putting the person first in the sentence structure.

**Response:** We have reviewed the manuscript and confirm this.

* Please review your manuscript and edit to ensure compliance with our inclusive language requirements (<https://journals.plos.org/plosmedicine/s/human-subjects-research#loc-categorization>).

**Response:** We have reviewed the manuscript and confirm this.

* Please consider including an acknowledgment of study participants and of individuals who played a role in data collection or participant care.

**Response:** We thank the editors for this suggestion. As this study is based on pseudonymized registry data, there were no individual participants, data collectors, or care providers directly involved in conducting this research; we therefore do not have specific individuals to acknowledge.

* Your study is observational and therefore causality cannot be inferred. Please remove language that implies causality and refer to associations instead (e.g. Abstract Conclusions or Author Summary).

**Response:** We have carefully reviewed the manuscript in its entirety and revised the wording throughout to remove causal interpretations and instead use descriptive and associational language when referring to our observed patterns.

ABSTRACT

* Please confirm that your abstract complies with our requirements, including providing all the information relevant to this study type <https://journals.plos.org/plosmedicine/s/submission-guidelines#loc-abstract>

**Response:** We have reviewed the abstract and confirm this.

* Please confirm that all numbers presented in the abstract are present and identical to numbers presented in the main manuscript text.

**Response:** We have reviewed the abstract and confirm this.

* In the last sentence of the Abstract Methods and Findings section, please describe the main limitation(s) of the study's methodology.

**Response:** We have added the main limitation of the study’s methodology as requested:

*“The main limitation of this study was statistical uncertainty for certain diagnostic groups due to small case numbers.”*

* Please quantify the results on incidence rates (with 95% CIs and p values).

**Response:** We have quantified the ASRs for overall childhood cancer in the Abstract. As these analyses are descriptive, p-values are not reported. We have opted not to include separate ASR estimates for each diagnostic group and pandemic year in the Abstract, as this would substantially increase its length and complexity and reduce readability. However, these results are fully reported in Table 2.

* Abstract Conclusions: Please revise with regard to using associational language.

**Response:** The section has been revised as advised and now reads:

*“Overall childhood cancer incidence and survival in Sweden showed no major changes during the COVID-19 pandemic. Fluctuations in diagnostic-group specific incidence may reflect delayed diagnoses or shifts in disease triggers; however, the timing of the ALL decline and the lack of AML rebound challenge this interpretation. Stable or improved survival suggests that any disruptions were not associated with poorer survival up to 1 year after diagnosis.”*

* Please specify which cancer types were included/excluded (e.g. “we included non-malignant central nervous system (CNS) tumors and intracranial/intraspinal germ cell tumors” as done in your Methods section).

**Response:** Childhood cancer was defined according to ICCC-3 criteria. We have therefore revised the first sentence of the Methods and Findings to read:

*“We conducted a nationwide, register-based cohort study including all children and adolescents (0–19 years) with a new cancer diagnosis,* ***defined according to the International Classification of Childhood Cancer, Third Edition (ICCC-3)****, reported to the Swedish National Cancer Register during 2015–2022 (N = 3,333; 2,069 pre-pandemic and 1,264 during the pandemic).”*

* In the abstract, please include the important dependent variables that are adjusted for in the analyses.

**Response:** We have revised the Abstract Methods and Findings section to explicitly state the variables adjusted for in the analyses, namely age at diagnosis, sex, and maternal education.

METHODS AND RESULTS

* Please remove ‘Role of the funding source’ from the Methods section. This information should only be included in the metadata in the online submission form.

**Response:** This section has now been removed.

* When discussing age, please add ‘years’ as a unit (including tables and figures).

**Response:** We have revised the manuscript to include “years” as the unit when reporting age throughout the text, tables, and figures.

* Figures: Please ensure to provide a label for the y-axis.

**Response:** We have revised all relevant figures to include a label for the y-axis, as requested.

* Figure 1: We agree with Reviewer #3 that the visualization of the information in Figure 1 could be improved, and we strongly recommend following their advice.

**Response:** We have revised Figure 1 in line with Reviewer #3 and editorial recommendations. Specifically, Figure 1 has been replaced by a variant of Figure S2, in which the individual years 2015-2019 are plotted using different symbols and shades of grey, and the 2015-2019 average is overlaid as a separate line. The pandemic years are also plotted separately, as before. As a result, Figure S2 has been removed from the Supplement, as it is now redundant.

* Table 2: For CNS tumors, you describe the difference between the 2015–2019 levels (47.0 (43.6–50.4)) and the 2020 levels (44.9 (37.1–52.6)) as comparable (a difference of 2.1). However, for Hodgkin, you describe a difference of 1.3 (with overlapping confidence interval (CI) values) as a decrease. Please explain how you determined whether there was a decrease, increase, or stable pattern.

**Response:** We thank the reviewers for spotting this unclarity. Our classification of patterns (increase, decrease, or stability) was based on descriptive comparisons of annual ASRs with the 2015–2019 average. For CNS tumors, we had not explicitly stated in the Results that while ASRs increased in 2020, they were below 2015–2019 levels in 2021, instead describing them as returning to pre-pandemic levels. We have now revised the relevant sentence in the Results to read:

*“For CNS tumors, ASRs increased in 2020 but returned to 2015–2019 levels* ***or below*** *in 2021 and remained stable in 2022”.*

* “Instead, there were indications of reduced 1-year mortality…” – based on the confidence intervals and the small n-numbers, we believe this is an overstatement. Please revise.

**Response:** We agree that the small number of events and wide confidence intervals preclude firm conclusions regarding mortality differences. We have therefore revised the relevant sentence to read:

*“Instead, there were indications,* ***although not statistically significant****, of reduced 1-year mortality among children diagnosed with ALL (aOR = 0.37; 95% CI [0.08,1.21]; p = 0.14) and CNS tumors (aOR = 0.61, 95% CI [0.31,1.14]; p = 0.13).”*

We further emphasize this limitation in the subsequent sentence:

*“These results were, however, based on only 5 and 15 deaths during 2020–2022, respectively.”*

* An exploratory analysis of 3-month mortality also suggested lower mortality following a CNS tumor diagnosis (aOR = 0.44, 95% CI [0.12–1.28]; p = 0.161) (S2 Figure). – please reference S3 Figure here instead and see our comment above.

**Response:** We thank the editors for highlighting the inconsistency in the referencing of the supplementary material. Following Reviewer #3’s and the editors’ recommendation, a variant of Figure S2 has been incorporated into the main manuscript as Figure 1 and is therefore no longer included in the Supplement. As a result, the referencing to S2 Figure in the highlighted sentence is now correct. We have also ensured that all tables and figures are referenced appropriately in the main text.

Moreover, we have revised the relevant sentence to read:

*“An exploratory analysis of 3-month mortality also suggested lower mortality following a CNS tumor diagnosis (aOR = 0.44, 95% CI [0.12,1.28]; p = 0.16),* ***although confidence intervals were wide*** *(S2 Figure).”*

* Please confirm that where relevant figures include 95% CIs.

**Response:** We have reviewed the Figures and confirm this.

* Please confirm you provided the unadjusted comparisons as well as the adjusted comparisons in all relevant Tables.

**Response:** We confirm that unadjusted comparisons are presented in S3 Figure.

* Please confirm you specified the variables controlled for in all relevant Tables.

**Response:** We have reviewed the Tables and confirm this.

DISCUSSION

* Due to the small number of cases and wide confidence intervals, we recommend carefully wording your findings and their interpretation (e.g., "Our findings rather suggest lower mortality...").

**Response:** We thank the editors for this suggestion. The noted sentence has been revised to: *“Our findings might rather suggest a small decrease in mortality among children diagnosed with ALL and CNS tumors in 2020–2022 compared with the pre-pandemic period, although estimates were imprecise.”*

In addition, we have reviewed the manuscript in its entirety and revised the wording where necessary to ensure a more cautious interpretation of the findings.

* Please remove the 'conclusions' subheading from the discussion. Please also remove any other subheadings from the discussion.

**Response:** All subheadings have been removed from the discussion.

General Editorial Requests

1) We ask every co-author listed on the manuscript to fill in a contributing author statement. If any of the co-authors have not filled in the statement, we will remind them to do so when the paper is revised. If all statements are not completed in a timely fashion this could hold up the re-review process. Should there be a problem getting one of your co-authors to fill in a statement we will be in contact. YOU MUST NOT ADD OR REMOVE AUTHORS UNLESS YOU HAVE ALERTED THE EDITOR HANDLING THE MANUSCRIPT TO THE CHANGE AND THEY SPECIFICALLY HAVE AGREED TO IT.

**Response:** All authors have filled in the statements.

2) Please ensure that the paper adheres to the PLOS Data Availability Policy (see <http://journals.plos.org/plosmedicine/s/data-availability>), which requires that all data underlying the study's findings be provided in a repository or as Supporting Information. For data residing with a third party, authors are required to provide instructions with contact information for obtaining the data. PLOS journals do not allow statements supported by "data not shown" or "unpublished results." For such statements, authors must provide supporting data or cite public sources that include it.

**Response:** We confirm the paper adheres to PLOS Data Availability Policy.

3) Please review your reference list to ensure that it is complete and correct. If you have cited papers that have been retracted, please include the rationale for doing so in the manuscript text, or remove these references and replace them with relevant current references. Any changes to the reference list should be mentioned in the rebuttal letter that accompanies your revised manuscript.

**Response:** We have reviewed the reference list as instructed.
